# Demographic Differences in Mortality in the District of Columbia

**DOI:** 10.1001/jamanetworkopen.2025.2290

**Published:** 2025-03-28

**Authors:** Maryam Hashemian, Katherine M. Conners, Jungnam Joo, Rebeka Rafi, Gretell Henriquez Santos, Joseph J. Shearer, Marcus R. Andrews, Tiffany M. Powell-Wiley, Meredith S. Shiels, Véronique L. Roger

**Affiliations:** 1 Heart Disease Phenomics Laboratory, Epidemiology and Community Health Branch, Division of Intramural Research, National Heart, Lung, and Blood Institute, National Institutes of Health, Bethesda, Maryland; 2 Office of Biostatistics Research, National Heart, Lung, and Blood Institute, National Institutes of Health, Bethesda, Maryland; 3 Social Determinants of Obesity and Cardiovascular Risk Laboratory, Cardiovascular Branch, National Heart, Lung, and Blood Institute, National Institutes of Health, Bethesda, Maryland; 4 National Institute on Minority Health and Health Disparities, National Institutes of Health, Bethesda, Maryland; 5 Infectious and Immunoepidemiology Branch, Division of Cancer Epidemiology and Genetics, National Cancer Institute, Rockville, Maryland

## Abstract

**Importance:**

Washington, District of Colombia (DC), has the largest gap in life expectancy between Black and White populations among major US cities.

**Objective:**

To investigate mortality, key modifiable cardiovascular disease (CVD) risk factors, and temporal trends for non-Hispanic Black and non-Hispanic White populations in Washington, DC, from 2000 to 2020.

**Design, Setting, and Participants:**

This cross-sectional study analyzed the Centers for Disease Control and Prevention Wide-Ranging Online Data for Epidemiologic Research database for mortality and the Behavioral Risk Factor Surveillance System for the prevalence of risk factors (obesity, hypertension, diabetes, smoking, and hypercholesterolemia) among Black and White populations in Washington, DC, from 2000 to 2020. All analyses were conducted in January 2024.

**Main Outcomes and Measures:**

All-cause and cause-specific, age-adjusted mortality rates per 100 000 person-years, prevalence of risk factors, and corresponding rate ratios (RRs) and 95% CIs comparing Black individuals to White individuals were assessed. Average annual percentage change (AAPC) was examined using joinpoint regression.

**Results:**

Among 102 710 deaths in Washington, DC (51 712 among males [50.3%], 26 100 among individuals aged ≥85 years [25.4%]; 82 308 among Black [80.1%] and 20 402 among White [19.9%] individuals), CVD (33 254 deaths [32.4%]) and cancer (22 677 deaths [22.1%]) accounted for more than half of deaths. All-cause mortality declined between 2000 and 2012 (AAPC, −2.6%; 95% CI, −4.5% to −1.9%), stagnated between 2012 and 2018, and increased between 2018 and 2020 (AAPC, 10.9%; 95% CI. 3.8% to 15.1%). CVD mortality declined between 2000 and 2011 (AAPC_,_ −3.1%; 95% CI, −4.3% to −2.4%) and plateaued thereafter in the Black population, contrasting with the monotonic decline in the White population from 2000 to 2020 (AAPC_,_ −4.7%; 95% CI, −5.3% to −4.1%), for a magnification of disparities from 2000 (RR, 1.5; 95% CI, 1.4 to 1.7) to 2020 (RR, 2.9; 95% CI, 2.5 to 3.3). Cancer mortality decreased from 2000 to 2020 but with a greater magnitude in the White (AAPC, −3.4%; 95% CI, −3.9% to −2.9%) than Black (AAPC_,_ −1.8%; 95% CI, −2.2% to −1.4%) population (RR for 2000, 1.6; 95% CI, 1.4 to 1.8 and RR for 2020, 2.1; 95% CI, 1.8 to 2.4). Risk factors were consistently more prevalent in the Black than White population (eg, hypertension: RR, 2.2; 95% CI, 1.8 to 2.7 in 2001 and 2.3; 95% CI, 1.9-2.6 in 2019). Disparities as assessed by RRs increased for smoking (AAPC, 4.3%; 95% CI, 3.8% to 5.6%), decreased for obesity (AAPC, −1.2%; 95% CI, −1.9% to −0.4%), and remained constant for diabetes, hypercholesterolemia, and hypertension.

**Conclusions and Relevance:**

In this study, all-cause, age-adjusted mortality was higher in the Black than White population, racial disparities worsened for CVD and cancer, and CVD risk factors were more prevalent in the Black population, underscoring the urgent need for precision public health interventions tailored toward high-risk populations.

## Key Points

**Question:**

Did mortality trends and cardiovascular disease (CVD) risk factors differ for the Black population compared with the White population in Washington, District of Columbia?

**Findings:**

In this cross-sectional study, all-cause mortality increased and CVD mortality stagnated in the Black population from 2011 to 2020, and disparities in CVD and cancer mortality worsened in the 2 decades studied. Racial disparities were observed for CVD risk factors and increased for smoking but decreased for obesity.

**Meaning:**

These findings suggest that CVD and cancer are major contributors to mortality disparities, and concurrent adverse trends in risk factors highlight the need for targeted interventions.

## Teaser

This cross-sectional study investigates disparities in mortality and cardiovascular disease risk factors between Black and White populations in Washington, District of Columbia, from 2000 to 2020.

## Introduction

After a century-long decline, all-cause mortality has stagnated in the US since 2015,^1^ particularly for cardiovascular diseases (CVDs), which remain the leading cause of death.^2,3,4,5,6^ All-cause, CVD, and cancer mortality have consistently been higher in the non-Hispanic Black population than the non-Hispanic White population,^7,8,9^ with all-cause mortality rates of 1119.0 vs 834.7 deaths per 100 000 individuals in the US in 2020.^10^

It is critical to examine city-level data because interventions and policy changes are most likely to occur locally.^11^ A 2021 cross-sectional study^12^ of mortality in the 30 most populous cities in the US reported that racial disparities in Washington, District of Columbia (DC), were the greatest. Building on these important observations, we undertook this study with the following goals: to evaluate trends in all-cause and cause-specific mortality in Washington, DC, from 2000 to 2020, highlight racial disparities, and concurrently examine trends in cardiovascular risk factors in the same population.

## Methods

### Study Setting and Population

In Washington, DC, non-Hispanic Black and non-Hispanic White residents constitute a similar fraction of the population (eFigure 1 in Supplement 1). The overall population refers to non-Hispanic Black (hereafter, *Black*) and non-Hispanic White (hereafter, *White*) populations. Presently, a distinct residential divide persists along an east-west axis between Black and White populations in the city. In 2000, 42% of the Black and 49% of the White population were male. The median age was 36 years in the Black population and 35 years in the White population.^13^ In 2020, 45% of the Black population and 49% of the White population were male. The median age was 36 years in the Black population and 34 years in the White population. The proportion of the uninsured population in 2004 was 10.2% for the White and 16.8% for the Black population, while in 2020 it was 1.8% in the White and 4.2% in the Black population in the city,^13^ below the national mean of 7.6% in the White and 9.9% in the Black population in 2020.^14^

### Mortality Trends

We used the Centers for Disease Control and Prevention (CDC) Wide-Ranging Online Data for Epidemiologic Research (CDC WONDER) dataset.^15^ CDC WONDER captures demographic characteristics and causes of death for all death certificates filed in the 50 states and Washington, DC. The underlying cause of death was categorized using the *International Statistical Classification of Diseases and Related Health Problems, Tenth Revision *(*ICD-10*). CVD was defined as heart disease and cerebrovascular disease. The World Health Organization defines the underlying cause of death as the disease or injury that leads directly to death.

### Risk Factors

We used data from the Behavioral Risk Factor Surveillance System (BRFSS).^16^ We examined the prevalence of CVD risk factors available from 2000 to 2020, including hypercholesterolemia, hypertension, diabetes, obesity (defined as a body mass index [calculated as weight in kilograms divided by height in meters squared] ≥30), and smoking among Washington, DC, adults.

Race and Hispanic ethnicity on death certificates are typically reported by the funeral director based on information from a next of kin or, if unavailable, by observation. In contrast, BRFSS race and ethnicity data are self-reported. The databases report the following race and ethnicity categories: American Indian or Alaska Native, Asian or Pacific Islander, Black or African American, Hispanic or Latino, not Hispanic or Latino, White, and not stated, don’t know, not sure, or refused. However, due to the limited sample size of other race and ethnicity groups in Washington, DC, our analysis was restricted to non-Hispanic Black and non-Hispanic White populations.

### Statistical Analysis

Deaths from January 1, 2000, to December 31, 2020, were categorized to identify the leading causes of death for individuals older than 14 years in Washington, DC. Using the 2000 US standard population, annual age-adjusted mortality rates (AAMRs) and 95% CIs were reported for Black and White populations, with corresponding rate ratios (RRs) comparing AAMRs for Black and White populations. For cause-specific mortality, the 2 most prevalent causes with sufficient cell size were reported in Black and White populations. Statistical analyses were conducted independently for CDC WONDER and BRFSS data using the same period when available. We used log-linear joinpoint regression models in Joinpoint Trend Analysis Software version 5.0.2 (National Cancer Institute)^17^ to calculate the average annual percentage change (AAPC) for AAMRs and CVD risk factor prevalence and corresponding RRs stratified by race. We used Joinpoint Trend Analysis Software that automates the selection process. It estimates joinpoints using the grid-search method to fit a regression model with unknown joinpoints and determines the number of joinpoints by a series of permutation tests that control the overall significance level.^18^ This approach addresses nonlinearity by allowing the model to fit piecewise linear segments. We used standard errors to calculate CIs using a Taylor series expansion technique.^19^ We conducted stratified analyses by 10-year age groups and sex. For stratified analyses by age, we report crude rates. Sensitivity analysis excluded deaths in 2020 to assess the association of the COVID-19 pandemic with our outcomes. This study used publicly available, deidentified CDC Wonder and BRFSS data, and so the National Heart, Lung, and Blood Institute, Division of Intramural Research determined that the study did not require ethical review or informed consent. The study is reported following the Strengthening the Reporting of Observational Studies in Epidemiology (STROBE) guideline. All statistical analyses were conducted using a 2-sided significance. Statistical significance was determined at *P* < .05. Data were analyzed in January 2024.

## Results

Between January 1, 2000, and December 31, 2020, there were 102 710 deaths in Washington, DC (82 308 among Black [80.1%] and 20 402 among White [19.9%] individuals; 51 712 among males [50.3%]; 26 100 among individuals aged ≥85 years [25.4%]). More than half of deaths were from CVD (33 254 deaths [32.4%]) or cancer (22 677 deaths [22.1%]) (Table 1).

**Table 1.** Leading Causes of Death

Cause of death (*ICD-10* code)	Deaths, No. (N = 102 710)^a^	Crude rate per 100 000	Age-adjusted rate per 100 000 (95% CI)
CVDs (I00-I09, I11, I13, I20-I51, I60-I69)	33 254	347.7	357.8 (353.9-361.7)
Cancer (C00-C97)	22 677	237.1	247.1 (243.9-250.3)
Accidents (unintentional injuries) (V01-X59, Y85-Y86)	5048	52.8	54.5 (53.0-56.0)
Diabetes (E10-E14)	3266	34.1	35.7 (34.4-36.9)
Chronic lower respiratory diseases (J40-J47)	2918	30.5	31.7 (30.6-32.9)
HIV disease (B20-B24)	2917	30.5	33.0 (31.8-34.2)
Assault (homicide) (U01-U02, X85-Y09, Y87.1)	2913	30.5	29.5 (28.4-30.6)
Alzheimer disease (G30)	2292	24.0	23.6 (22.6-24.6)
Septicemia (A40-A41)	1865	19.5	20.3 (19.4-21.2)
Influenza and pneumonia (J09-J18)	1675	17.5	17.9 (17-18.7)
Nephritis, nephrotic syndrome, and nephrosis (N00-N07, N17-N19, N25-N27)	1320	13.8	14.3 (13.5-15.1)
Essential hypertension and hypertensive renal disease (I10, I12, I15)	1317	13.8	14.1 (13.4-14.9)
Chronic liver disease and cirrhosis (K70, K73-K74)	1092	11.4	11.9 (11.2-12.6)
COVID-19 (U07.1)	709	7.4	7.6 (7.1-8.2)

Abbreviations: CVD, cardiovascular disease; *ICD-10*, *International Statistical Classification of Diseases and Related Health Problems, Tenth Revision*.

^a^ Deaths are given by *ICD-10* code in Washington, District of Columbia, from 2000 to 2020 among a total population of 11 037 546 individuals.

### Mortality in Overall Population and Temporal Trends

#### All-Cause Mortality

The all-cause AAMR in the overall population followed a pattern of abrupt decline (AAPC for 2000-2012, −3.0%; 95% CI, −5.0% to −0.8%), followed by a plateau (AAPC for 2012-2018, −0.7%; 95% CI. −3.5% to 1.3%) and an increase after 2018, likely reflecting the association of the COVID-19 pandemic in 2020 with outcomes (AAPC for 2018-2020, 7.9%; 95% CI, 1.5% to 11.6%) (Table 2). The all-cause AAMR in the overall population decreased from 1372.4 deaths (95% CI, 1336.8 to 1408.0 deaths) per 100 000 individuals in 2000 to 1136.9 deaths (95% CI, 1107.0 to 1166.8deaths) per 100 000 individuals in 2020 (AAPC for 2000-2020, −1.3%; 95% CI, −1.7% to −1.0%).

**Table 2.** All-Cause and Cause-Specific Mortality Rates and Percentage Change by Race

Cause of mortality	AAPC, % (95% CI)		AAMR, No./100 000 (95% CI)	
	For time segment	2000 to 2020	In 2000	In 2020
**All-cause**				
Overall				
2000 to 2012	−3.0 (−5.0 to −0.8)	−1.3 (−1.7 to −1.0)	1372.4 (1336.8 to 1408.0)	1136.9 (1107.0 to 1166.8)
2012 to 2018	−0.7 (−3.5 to 1.3)			
2018 to 2020	7.9 (1.5 to 11.6)			
Black^a^				
2000 to 2012	−2.6 (−4.5 to −1.9)	−0.4 (−0.9 to −0.1)	1595.8 (1549.4 to 1642.3)	1583.3 (1537.1 to 1629.5)
2012 to 2018	0.4 (−2.4 to 2.8)			
2018 to 2020	10.9 (3.8 to 15.1)			
White, 2000 to 2020^b^	−3.3 (−3.7 to −2.9)	−3.3 (−3.7 to −2.9)	897.3 (845.4 to 949.2)	491.7 (460.5 to 522.9)
**CVD**				
Overall				
2000 to 2011	−3.7 (−4.9 to −3.1)	−2.5 (−2.9 to −2.2)	464.4 (443.7 to 485)	303.5 (288.1 to 318.8)
2011 to 2020	−1.1 (−2.0 to 0.8)			
Black^a^				
2000 to 2011	−3.1 (−4.3 to −2.4)	−1.6 (−2.0 to −1.3)	519.7 (493.4 to 546.1)	409 (386 to 432.1)
2011 to 2020	0.1 (−0.9 to 2.1)			
White, 2000 to 2020^b^	−4.7 (−5.3 to −4.1)	−4.7 (−5.3 to −4.1)	337.3 (305.8 to 368.9)	141.1 (124.5 to 157.8)
**Cancer**				
Overall				
2000 to 2020	−2.4 (−2.7 to −2.1)	−2.4 (−2.7 to −2.1)	317.4 (300.3 to 334.6)	186.9 (174.8 to 199.1)
Black, 2000 to 2020^a^	−1.8 (−2.2 to −1.4)	−1.8 (−2.2 to −1.4)	356.7 (334.9 to 378.5)	234.4 (216.9 to 251.9)
White, 2000 to 2020^b^	−3.4 (−3.9 to −2.9)	−3.4 (−3.9 to −2.9)	228.7 (201.8 to 255.6)	111.9 (96.8 to 126.9)

Abbreviations: AAMR, age-adjusted mortality rate; AAPC, average annual percent change; CVD, cardiovascular disease.

^a^ Black denotes the non-Hispanic Black population.

^b^ White denotes the non-Hispanic White population.

#### Cause-Specific Mortality

The leading underlying causes of death were CVD, cancer, accidents (5048 deaths [4.9%]), diabetes (3266 deaths [3.2%]), and chronic lower respiratory disease (2918 deaths [2.8%]) (Table 1). We focused the analysis on deaths from CVD and cancer given that they accounted for more than half of deaths. Corresponding temporal trends were heterogeneous (Table 2). Cancer mortality declined monotonically from 2000 to 2020 (AAPC, −2.4%; 95% CI, −2.7% to −2.1%), while CVD mortality declined from 2000 to 2011 (AAPC, −3.7%; 95% CI, −4.9% to −3.1%) and stabilized from 2011 to 2020 (AAPC_,_ −1.1%; 95% CI, −2.0% to 0.8%).

### Mortality by Race and Temporal Trends

#### All-Cause Mortality

In the White population, all-cause mortality decreased from 897.3 deaths (95% CI, 845.4 to 949.2 deaths) per 100 000 persons in 2000 to 491.7 deaths (95% CI, 460.5 to 522.9 deaths) per 100 000 persons in 2020 (Table 2). The trend exhibited a monotonic decline from 2000 to 2020 (AAPC, −3.3%; 95% CI. −3.7% to −2.9%). In the Black population, all-cause mortality rates decreased from 2000 to 2012 (AAPC, −2.6%; 95% CI, −4.5% to −1.9%), stagnated from 2012 to 2018 (AAPC_,_ 0.4%; 95% CI, −2.4% to 2.8%), and then increased from 2018 to 2020 (AAPC, 10. 9%; 95% CI, 3.8% to 15.1%) (Table 2). From 2000 to 2020, mortality rates declined substantially less in the Black population (AAPC_,_ −0.4%; 95% CI. −0.9% to −0.1%) than the White population (AAPC_,_ −3.3%; 95% CI, −3.7% to −2.9%) (Table 2) for a magnification of disparities over time.

#### Cause-Specific Mortality

CVD mortality declined monotonically in the White population from 2000 to 2020 (AAPC_,_ −4.7%; 95% CI, −5.3% to −4.1%) (Table 2), in contrast to the Black population, among whom mortality rates declined between 2000 and 2011 (AAPC_,_ −3.1%; 95% CI, −4.3% to −2.4%) and plateaued from 2011 to 2020 (AAPC_,_ −0.1%; 95% CI, −0.9% to 2.1%), for a magnification of disparities from 2000 (RR, 1.5; 95% CI, 1.4 to 1.7) to 2020 (RR, 2.9; 95% CI, 2.5 to 3.3). The pattern was ostensibly different for cancer mortality, which decreased over time for Black and White populations (Table 2). However, there was a greater magnitude of decline from 2000 to 2020 in the White population (AAPC, −3.4%; 95% CI, −3.9% to −2.9%) than the Black population (AAPC_,_ −1.8%; 95% CI, −2.2% to −1.4%), for a magnification of disparities (RR for 2000, 1.6; 95% CI, 1.4 to 1.8 vs RR for 2020, 2.1; 95% CI, 1.8 to 2.4).

**Figure 1.**
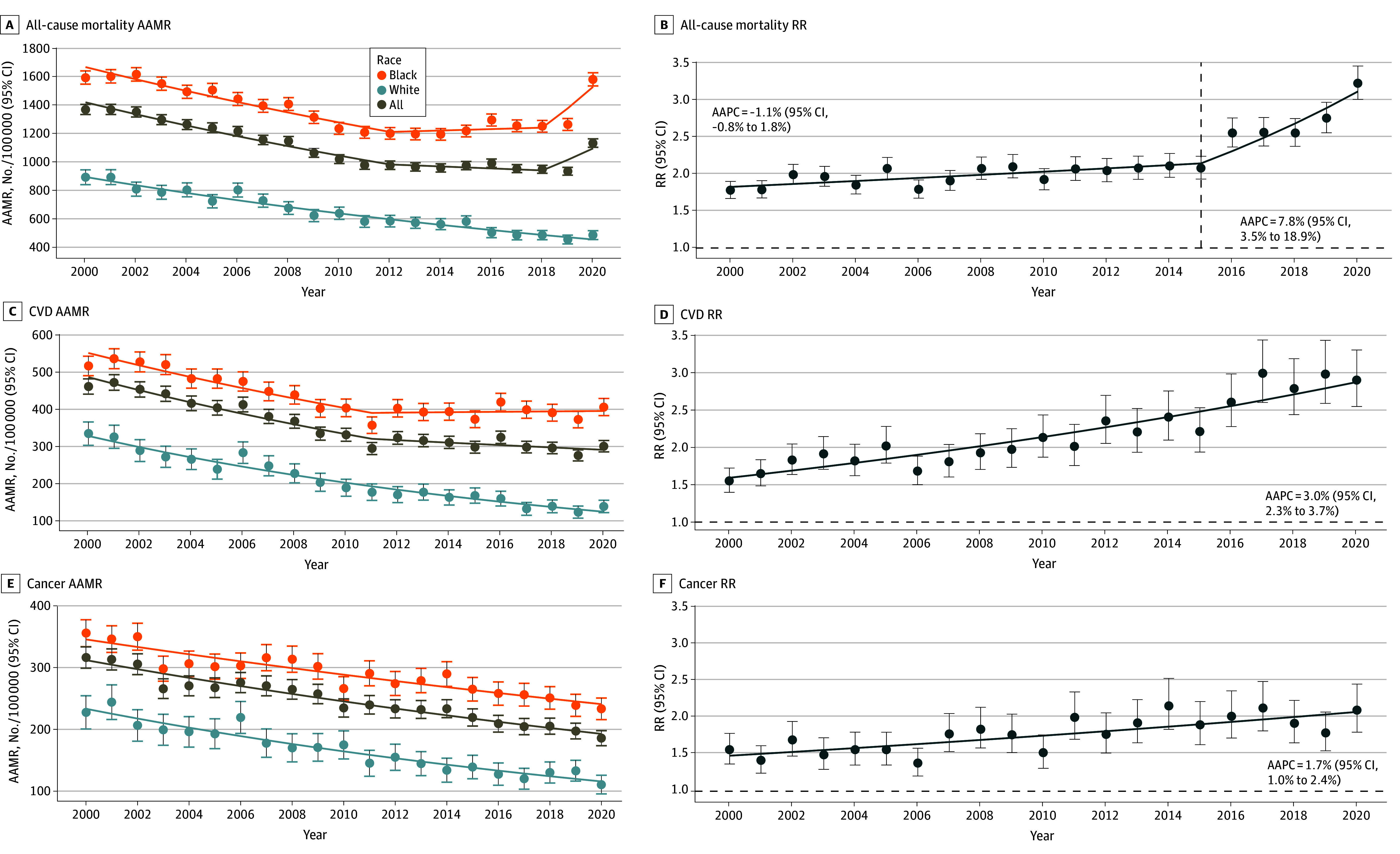
All-Cause and Cause-Specific Mortality by Race Age-adjusted mortality rates (AAMRs) expressed per 100 000 individuals (A) and rate ratios (RRs) comparing Black individuals to White individuals are given in Washington, District of Columbia, from 2000 to 2020. AAPC indicates average annual percentage change; CVD, cardiovascular disease; horizontal dashed lines, RR reference = 1.

AAMRs from all-cause and cause-specific mortality were consistently higher in the Black population (Figure 1A), as also shown by RRs comparing death rates in Black compared with White populations (Figure 1B). The magnitude of these disparities as assessed by RRs increased significantly between 2015 and 2020 for all-cause mortality (AAPC_,_ 7.8%; 95% CI, 3.5%-18.9%) and from 2000 to 2020 for CVD (AAPC_,_ 3.0%; 95% CI, 2.3%-3.7%) and cancer mortality (AAPC_,_ 1.7%; 95% CI, 1.0%-2.4%). In 2020, COVID-19 was listed as a new cause of death, with an excess risk of deaths in the Black vs White population (RR, 4.1; 95% CI, 3.3-5.1).

### Mortality by Race and Sex With Temporal Trends

#### All-Cause Mortality

**Figure 2.**
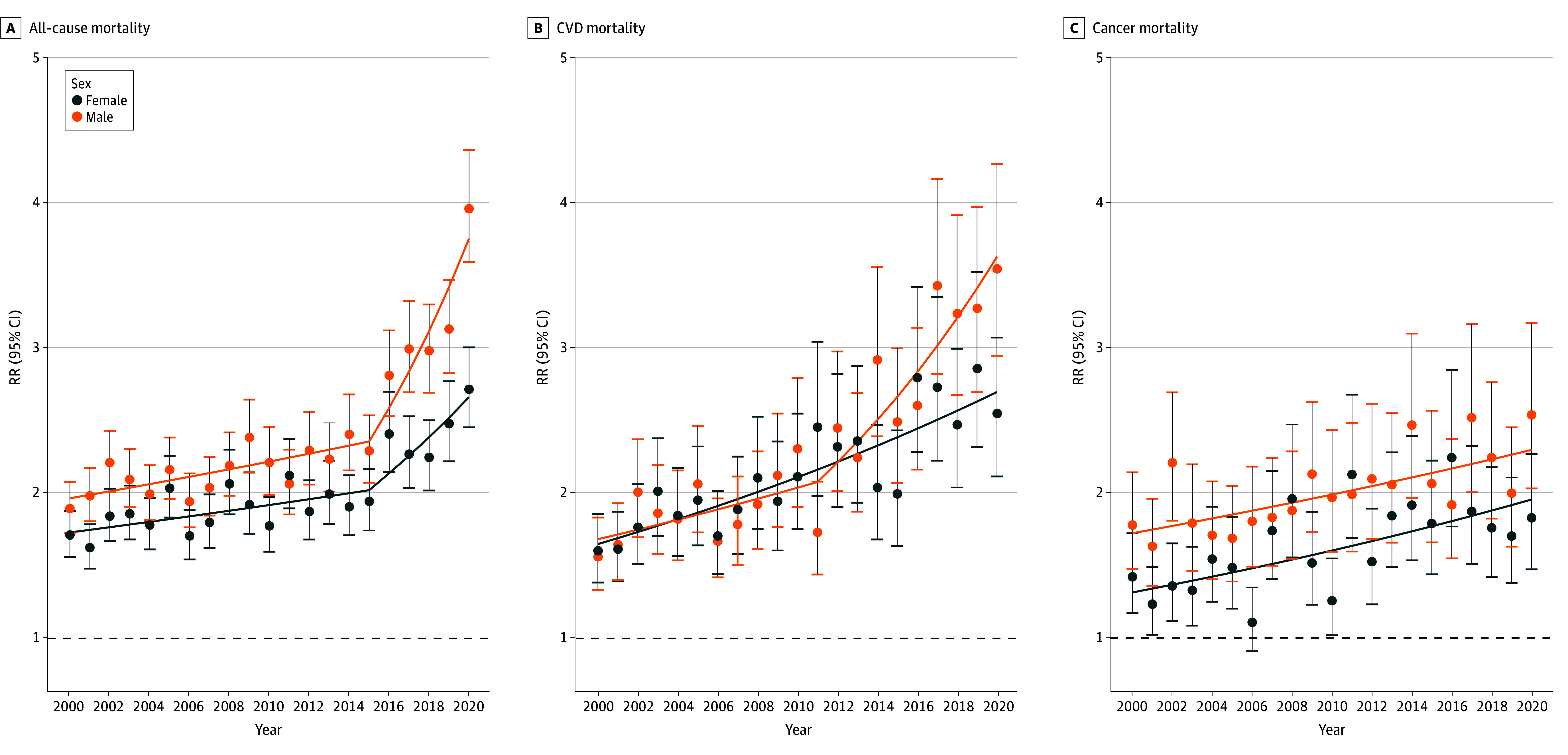
Rate Ratios (RRs) for Mortality Comparing Black Individuals to White Individuals by Sex RRs are given from 2000 to 2020 for all-cause mortality (A), cardiovascular disease (CVD) mortality (B), and cancer mortality (C). Horizontal dashed lines indicate RR reference = 1.

RRs increased over time for both sexes, with more pronounced increases from 2015 to 2020 among males (AAPC_,_ 9.7%; 95% CI, 5.2%-21.7%) than females (AAPC_,_ 5.6%; 95% CI, 1.9%-15.4%) (Figure 2). In 2020, the RR for all-cause mortality comparing Black males to White males was 4.0 (95% CI, 3.8-4.2) vs 2.7 (95% CI, 2.6-2.9) comparing Black females to White females. This was the highest RR observed for both sexes during the study period.

#### Cause-Specific Mortality

RRs increased for CVD deaths in males from 2011 to 2020 (AAPC_,_ 6.4%; 95% CI, 3.2%-20.3%) and for cancer deaths from 2000 to 2020 in females (AAPC_,_ 2.0%; 95% CI, 0.8%-3.1%) and males (AAPC_,_ 1.4%; 95% CI, 0.7%-2.2%). RRs for COVID-19 deaths were more prominent among males (RR, 4.7; 95% CI, 3.5-6.3) than females (RR, 4.0; 95% CI, 2.9-5.6).

### Mortality by Ward After the Pandemic

In 2020, CVD, cancer, and COVID-19 were the top 3 leading causes of death in Washington, DC. CVD was the top cause of death in all 8 wards, and COVID-19 was the second leading cause of death in wards 1 and 8 (eFigure 2 in Supplement 1).^20^

### Risk Factors by Race and Temporal Trends

**Figure 3.**
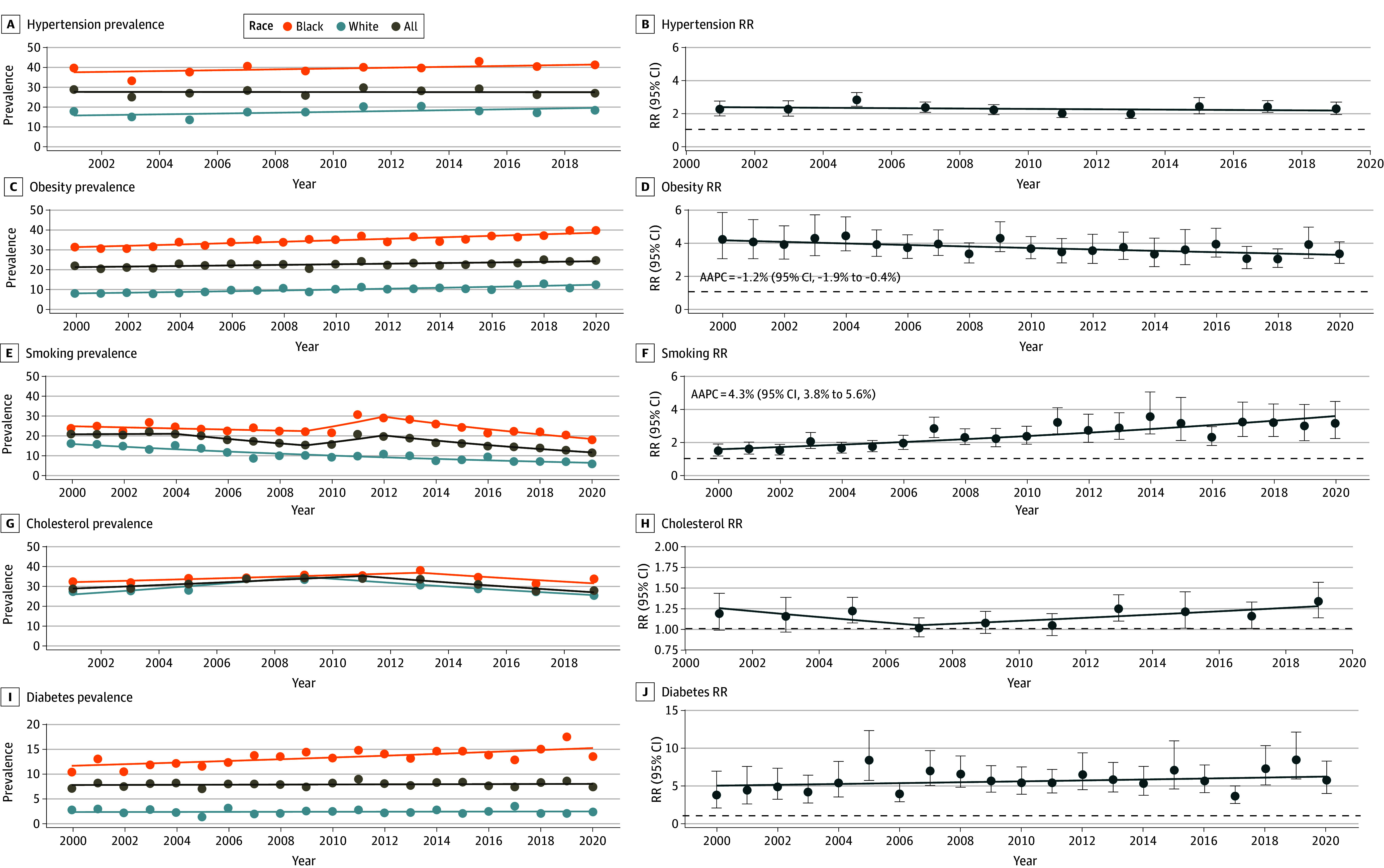
Cardiovascular Disease Risk Factors by Race Prevalence (A) and rate ratios (RRs) of risk factors comparing Black individuals to White individuals (B) are given from 2000 to 2020 using Behavioral Risk Factor Surveillance System survey data. Data were not available for the entire study period for high cholesterol levels and hypertension. AAPC (average annual percentage change) is shown only for significant values. Horizontal dashed lines indicate RR reference = 1.

Using BRFSS data, we examined the prevalence of key modifiable CVD risk factors, their associated temporal trends between 2000 and 2020 when available, and prevalence ratios comparing Black individuals to White individuals (Figure 3). Hypertension, obesity, smoking, and diabetes were more prevalent in the Black population than the White population (Figure 3A). Throughout the 2 decades, hypertension remained the most prevalent risk factor in the Black population (40.0% [95% CI, 36.2%-43.8%] of individuals in 2001 and 41.6% [95% CI, 38.3%-45.0%] of individuals in 2019) compared with the White population (17.9% [95% CI, 14.7%-21.1%] of individuals in 2001 and 18.4% [95% CI, 15.7%-21.1%] of individuals in 2019) and was consistently 2 times more prevalent in the Black population (RR, 2.2; 95% CI, 1.8-2.7 in 2001 and RR, 2.3; 95% CI, 1.9-2.6 in 2019) (Figure 3B); the AAPC for 2001 to 2019 was not significant. Obesity was more prevalent in the Black population (31.1% [95% CI, 27.6%-34.6%] of individuals in 2000 and 39.6% [95% CI, 35.9%-43.3%] of individuals in 2020) compared with the White population (7.4% [95% CI, 5.1%-9.7%] of individuals in 2000 and 11.9% [95% CI, 9.8%-13.9%] of individuals in 2020) over the 2 decades. Given the more rapid increase in the White population, there was a decrease in the prevalence RR from 2000 (RR, 4.2; 95% CI, 2.8-5.6) to 2020 (RR, 3.3; 95% CI, 2.7-3.9), for an AAPC from 2000 to 2020 of −1.2% (95% CI, −1.9% to −0.4%). Smoking prevalence decreased from 2000 to 2020 in Black (23.8% [95% CI, 20.6%-27.0%] of individuals in 2000 vs 18.0% [95% CI, 15.2%-20.8%] of individuals in 2020) and White (16.1% [95% CI, 12.8%-19.4%] of individuals in 2000 vs 5.7% [95% CI, 3.9%-7.5%] of individuals in 2020) populations. Given the more rapid decrease in the White population, disparities were amplified from 2000 (RR, 1.5; 95% CI, 1.2-1.9) to 2020 (RR, 3.2; 95% CI, 2.1- 4.3), for an AAPC from 2000 to 2020 of 4.3% (95% CI, 3.8 %-5.6%). Diabetes prevalence was also higher in the Black population (10.5% [95% CI, 8.2%-12.8%] of individuals in 2000 and 13.7% [95% CI, 11.5%-15.8%] of individuals in 2020) compared with the White population (2.8% [95% CI, 1.2%-4.4%] of individuals in 2000 and 2.4% [95% CI, 1.6%-3.2%] of individuals in 2020); however, RRs remained constant from 2000 (RR_, _3.8; 95% CI, 1.5-6.0) to 2020 (RR_,_ 5.7; 95% CI, 3.6-7.8); the AAPC from 2000 to 2020 was not significant.

### Sensitivity Analyses

We repeated the analyses after excluding deaths that occurred in 2020 (eFigures 3-5 in Supplement 1), and the increase in all-cause mortality rates after 2018 was no longer observed in the Black or overall populations (eFigure 3 in Supplement 1). However, results for CVD and cancer mortality remained unchanged (eFigure 3 in Supplement 1). Similarly, disparities as assessed by RRs comparing the Black population to the White population for all-cause mortality decreased (AAPC for 2015-2020, 7.8%; 95% CI, 3.5%-18.9% vs AAPC for 2014-2019, 5.7%; 95% CI, 2.3%-14.5%), but there was no change for CVD or cancer mortality (eFigure 4 in Supplement 1). The decrease in RRs occurred in makes and females for all-cause mortality but not CVD or cancer mortality (eFigure 5 in Supplement 1).

We used an autocorrelation analysis to evaluate whether serial correlation was present in our time-series data. Results were mostly similar, supporting the robustness of our joinpoint results (eFigure 6 in Supplement 1).

## Discussion

This cross-sectional study provides new insights into trends in mortality and key CVD risk factors in Washington, DC, over the past 2 decades. We found a 3-phase evolution in all-cause AAMR: a significant decline during 2000 to 2012, stagnation during 2012 to 2018, and an increase during 2018 to 2020, reflecting outcomes associated with the COVID-19 pandemic. The leading causes of death were CVD and cancer. While both causes of death declined in the overall population during 2000 to 2020, the Black population experienced higher AAMRs from all causes, CVD, and cancer, which increased over time. Obesity, hypertension, diabetes, and smoking were more prevalent in the Black population. Only smoking declined over time in both groups, and disparities decreased for obesity.

### Mortality Trends and Disparities

Given decreases in mortality in the White population since 2000, paired with increased mortality in the Black population since 2012, there was a widening of the Black-White mortality gap in Washington, DC. This finding was consistent with results from previous studies.^21,22^

The inflection point indicating the shift in directionality in all-cause mortality was consistent with the inflection point for CVD mortality in 2011. Profound differences in CVD mortality between Black and White populations increased over time, especially after 2011, several years before the COVID-19 pandemic. Our findings align with national data indicating that the decline in CVD slowed significantly after 2011.^2,23^ Notably, these disparities widened during a period marked by advancements in heart disease treatment and prevention. Decreases in smoking, along with advances in early detection and treatment have been associated with a steady decline in cancer mortality rate in the US, which was also experienced by residents of Washington, DC^24^; however, we observed increasing disparities over time.

These disparities were observed in the context of adverse structural social and economic factors.^12^ Presently, a distinct residential divide persists along an east-west axis in Washington, DC, between Black and White populations. Such segregation is linked to suboptimal schools and educational opportunities and the existence of areas of limited economic investment, inadequate infrastructure, limited access to healthy foods, health care infrastructure, increased exposure to environmental hazards, and higher crime rates.^25,26^ Interventions to ensure equitable access to quality education, employment opportunities, secure neighborhoods, and affordable housing will be critical to reducing health disparities in Washington, DC.^22^ While health care is critical in this context, it is important to underscore that only 4.2% of the Black population of Washington, DC, is uninsured.^14^ Hence, it stands to reason that advancements in the prevention and treatment of chronic diseases have not benefitted all populations equally despite widespread health insurance coverage. This is in keeping with the Link and Phelan fundamental cause theory,^27^ which highlights that health disparities persist when structural determinants, including systemic racism and inequities in social resources, are not addressed. Reasons for these enduring disparities are complex and encompass factors ranging from individual-level to societal influences according to the National Institute on Minority Health and Health Disparities framework.^28,29^ It emphasizes the need for interventions tailored toward structural components of health.^27^

A demographic shift may be contributing to widening Black-White longevity disparities in Washington, DC. Between 2000 and 2020, the White population increased from 30% to 40%, accompanied by an increasing socioeconomic gap. In 2023, the median household income for the Black population was $54 000, compared with $162 000 for the White population.^30^ Education, a crucial social determinant of health,^31^ influences these disparities; 90% of the White population held a bachelor’s degree or higher in 2020, compared with 29% of Black residents.^32^ The influx of a White population that was wealthier and more highly educated, alongside increasing socioeconomic disparities, likely contributed to the mortality gap.

Observed disparities may also arise from the high prevalence of CVD risk factors, including hypertension, obesity, type 2 diabetes, and smoking, in the Black population in Washington, DC, consistent with prior studies in the US.^28^ Reductions in major risk factors may account for approximately half of the decrease in US deaths from coronary disease during 1980 to 2000.^33^ These findings suggest that implementing public health interventions citywide is crucial, especially among the Black population in Washington, DC.

All-cause mortality was greater among Black males, consistent with US data, which additionally shows greater disparity for younger age groups.^34^ The recent decline in US life expectancy reflects increasing AAMRs among young and middle-aged adults,^35^ delineating prevention opportunities to reach higher-risk groups. Building on promising results from pilot studies,^36,37^ there is a need for more community-engaged interventions aimed at CVD risk factors in Black males. These may leverage models like the Diabetes Prevention Program^38^ and the American Hearth Association Check, Change, Control blood pressure self-management program^36^ and incorporate technologies such as wearable mobile health devices.^37^ Such approaches could enhance health outcomes in resource-limited communities and across diverse populations.^37^ Given the variability in ward-level resources in Washington, DC, with particularly low levels of internet access in wards 7 and 8,^39^ future interventions should account for infrastructure disparities when designing technology-dependent strategies.

When we exclude 2020 from our analyses, RRs comparing Black individuals to White individuals for AAMR decreased. This finding aligns with the amplification of racial disparities in Washington, DC, during the COVID-19 pandemic,^40,41^ which exposed the breadth and depth of health disparities in the US.^35^ Populations at increased risk, including individuals with lower socioeconomic status, may have been disproportionately affected by COVID-19 or disruptions in the care of other diseases.^42^ Expanding community-focused strategies, especially with targeted interventions at the ward level, is important for reducing health disparities in Washington, DC.^43^ Community health workers, the largest group in the health workforce, are essential for reaching at-risk groups. Integrating social support into primary care may strengthen these efforts.^44^ Local leaders can support the expansion of community-focused strategies by promoting teamwork, building trust, and encouraging community involvement to improve health outcomes.^45^

### Limitations

This study has several limitations that should be acknowledged. Misclassification of cause of death or race assignment was possible^46^ but unlikely to have significantly affected our findings over time. Due to small numbers, we could not carry out the same analysis in the Latino or Hispanic population, which makes up approximately 11% of the city’s population and a higher percentage in specific wards (19% in ward 1 and 23% in ward 4).^20^ Including this group would further enhance the generalizability of our findings. Joinpoint analysis can be sensitive to the number of deaths, leading to labeling minor changes as inflection points for a high number of deaths, and may fail to identify joinpoints for smaller counts. While joinpoint analysis is effective at modeling nonlinear trends, it may have limitations for highly curved or complex nonlinear functions. We acknowledge the potential influence of autocorrelation on the sensitivity of joinpoint analysis; however, repeated analyses using autocorrelated errors yielded similar outcomes, supporting the robustness of our findings. The observed increase in CVD mortality could stem from shifts in CVD incidence, case fatality, or both, although national data on the incidence of CVD is scarce.^47^

## Conclusions

This serial cross-sectional study highlighted increased disparities in all-cause and cause-specific mortality in Washington, DC, over the past 2 decades, with CVD and cancer as major contributors. Adverse trends in risk factors and ward-level data further point to areas for intervention. Our study offers robust insights into all-cause and cause-specific mortality, risk factors, and their temporal evolution, highlighting evolving disparities between Black and White populations. City level analyses are important given that policy change is more likely to occur locally. Our findings emphasize the urgent need for change and provide insights for tailored interventions, particularly those addressing social determinants of health, in line with precision public health principles.^48^

## Notes

Supplement 1. **eFigure 1.** Race and ethnicity distribution in the District of Columbia **eFigure 2.** Leading causes of death in the District of Columbia by ward in 2020 **eFigure 3.** Age-adjusted mortality rate in Black, White, and overall population before and after excluding deaths in 2020 **eFigure 4.** Rate ratios comparing Black individuals to White individuals before and after excluding deaths in 2020 **eFigure 5.** Rate ratios comparing Black individuals to White individuals by sex before and after excluding deaths in 2020 **eFigure 6.** Joinpoint analysis considering autocorrelated errors for age-adjusted mortality rate in Black, White, and overall population after excluding deaths in 2020

Supplement 2.
Data Sharing Statement
