# Supplementary material for: Demographic Differences in Mortality in the District of Columbia
Source: JAMA Netw Open. 2025 Mar 28;8(3):e252290. doi: 10.1001/jamanetworkopen.2025.2290 (PMC11953761; doi:10.1001/jamanetworkopen.2025.2290)
Supplement: Supplement 1. — eFigure 1. Race and ethnicity distribution in the District of Columbia eFigure 2. Leading causes of death in the District of Columbia by ward in 2020 eFigure 3. Age-adjusted mortality rate in Black, White, and overall population before and after excluding deaths in 2020 eFigure 4. Rate ratios comparing Black individuals to White individuals before and after excluding deaths in 2020 eFigure 5. Rate ratios comparing Black individuals to White individuals by sex before and after excluding deaths in 2020 eFigure 6. Joinpoint analysis considering autocorrelated errors for age-adjusted mortality rate in Black, White, and overall population after excluding deaths in 2020 [file jamanetwopen-e252290-s001.pdf]

Supplemental Online Content

Hashemian M, Conners KM, Joo J, et al. Racial disparities in mortality in the District of Columbia. *JAMA Netw Open*. 2025;8(3):e252290. doi:10.1001/jamanetworkopen.2025.2290

eFigure 1. Race and ethnicity distribution in the District of Columbia

eFigure 2. Leading causes of death in the District of Columbia by ward in 2020

eFigure 3. Age-adjusted mortality rate in Black, White, and overall population before and after excluding deaths in 2020

eFigure 4. Rate ratios comparing Black individuals to White individuals before and after excluding deaths in 2020

eFigure 5. Rate ratios comparing Black individuals to White individuals by sex before and after excluding deaths in 2020

eFigure 6. Joinpoint analysis considering autocorrelated errors for age-adjusted mortality rate in Black, White, and overall population after excluding deaths in 2020

This supplemental material has been provided by the authors to give readers additional information about their work.

© 2025 Hashemian M et al. *JAMA Network Open*.

eFigure 1. Race and Ethnicity Distribution in the District of Columbia (A) by year, (B) by Ward in 2020

(A)

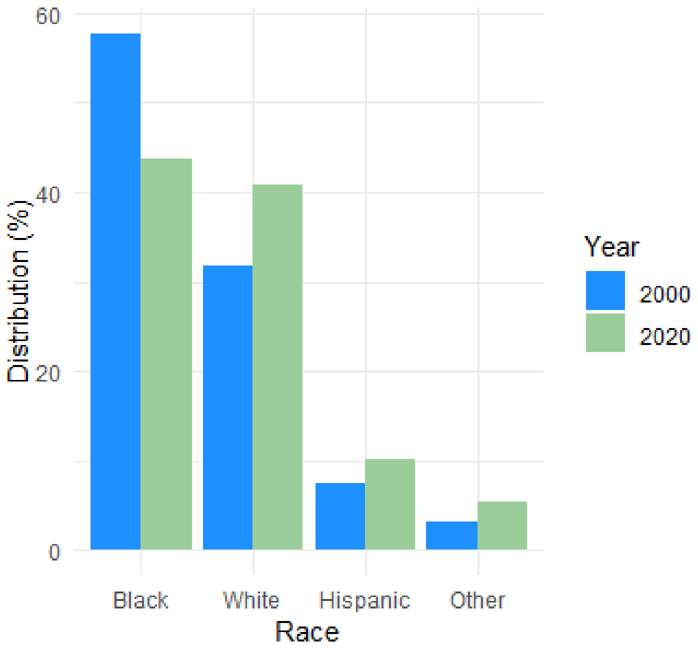

(B)

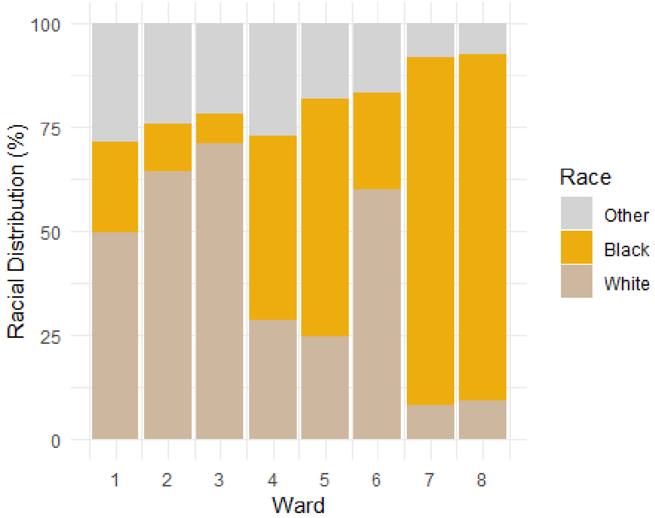

*Other: Hispanic or Latino, Asian, American Indian or Alaska Native, Native Hawaiian or other Pacific Islander, Other race, and Multiracial. Hispanics/Latinos can be of any race.

© 2025 Hashemian M et al. *JAMA Network Open*.

eFigure 2. The leading causes of death in the District of Columbia by Ward in 2020

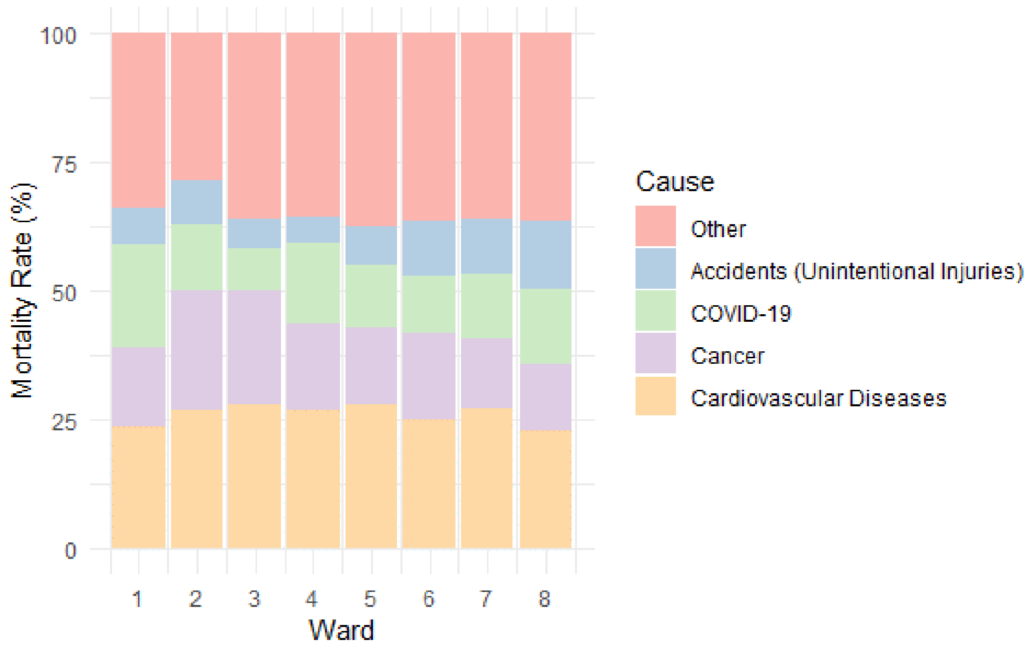

© 2025 Hashemian M et al. *JAMA Network Open*.

eFigure 3, Age-adjusted mortality rate in Black, White, and overall population before and after excluding deaths occurred in 2020

(A) All-cause mortality before (left) and after (right) excluding 2020

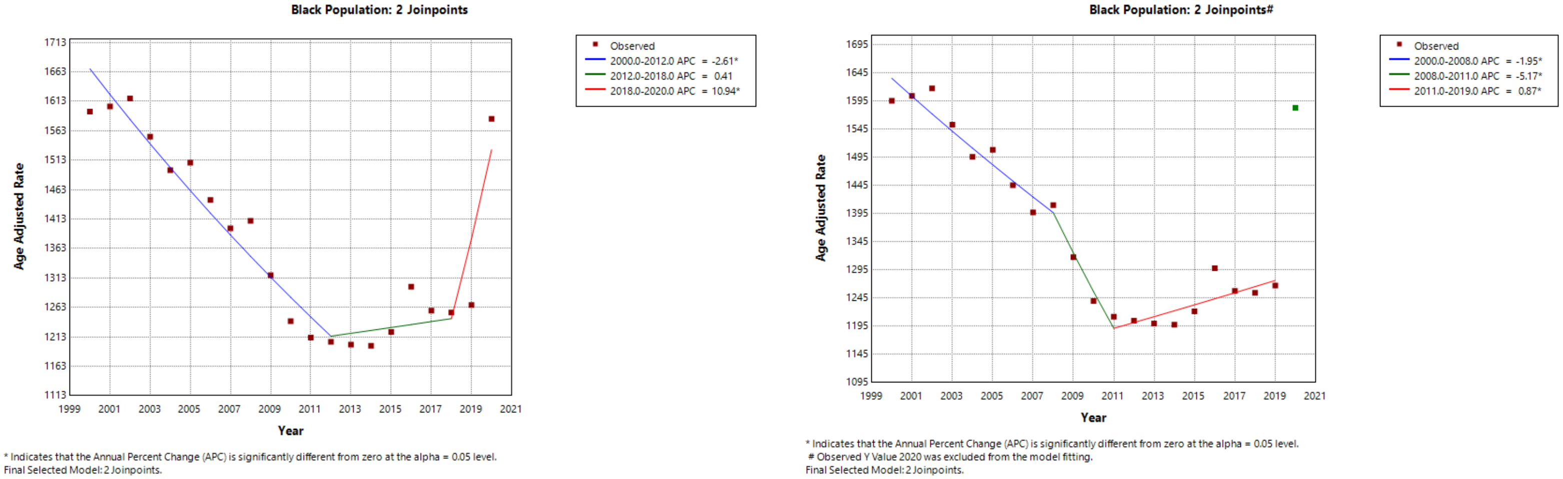

© 2025 Hashemian M et al. *JAMA Network Open*.

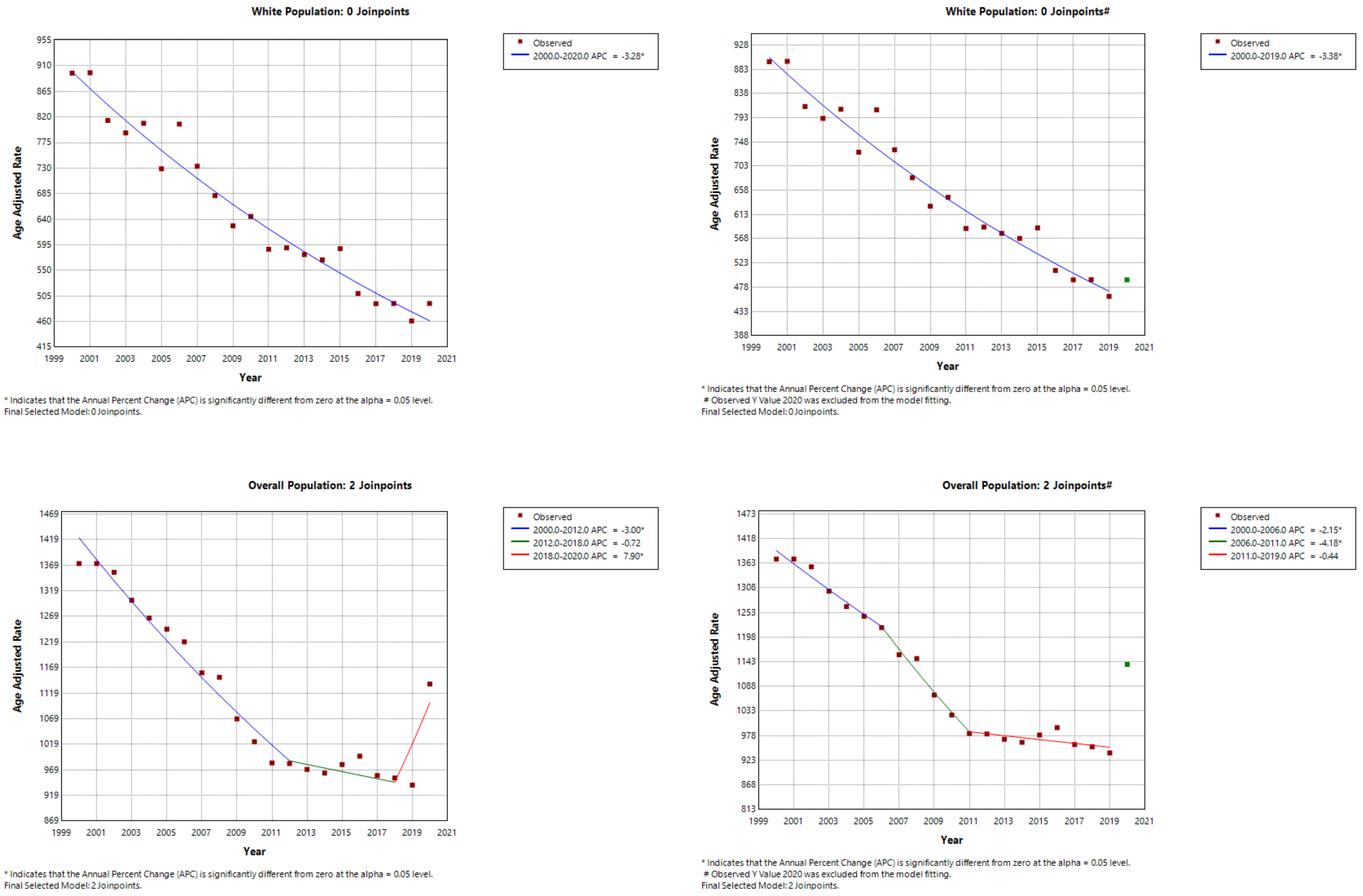

(B) CVD mortality before (left) and after (right) excluding 2020

© 2025 Hashemian M et al. *JAMA Network Open*.

Black Population: 1 Joinpoint

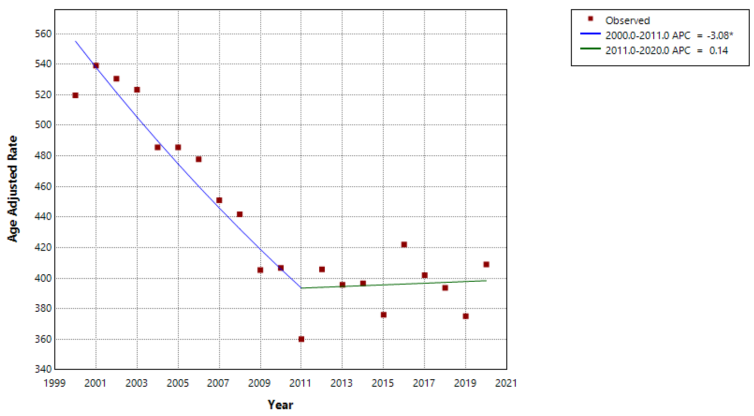

* Indicates that the Annual Percent Change (APC) is significantly different from zero at the alpha = 0.05 level.
Final Selected Model: 1 Joinpoint.

Black Population: 1 Joinpoint#

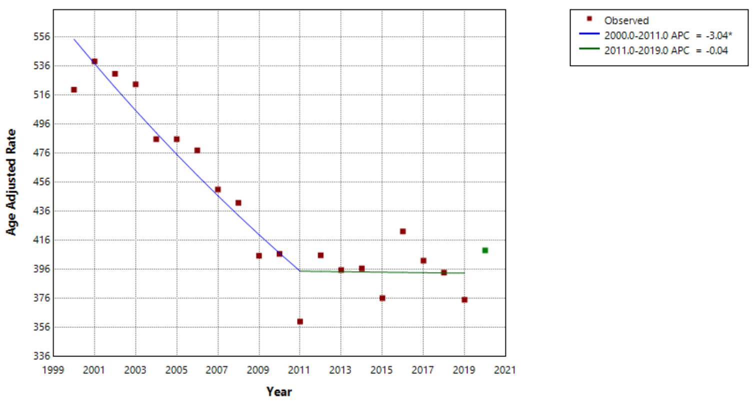

* Indicates that the Annual Percent Change (APC) is significantly different from zero at the alpha = 0.05 level.
# Observed Y Value 2020 was excluded from the model fitting.
Final Selected Model: 1 Joinpoint.

White Population: 0 Joinpoints

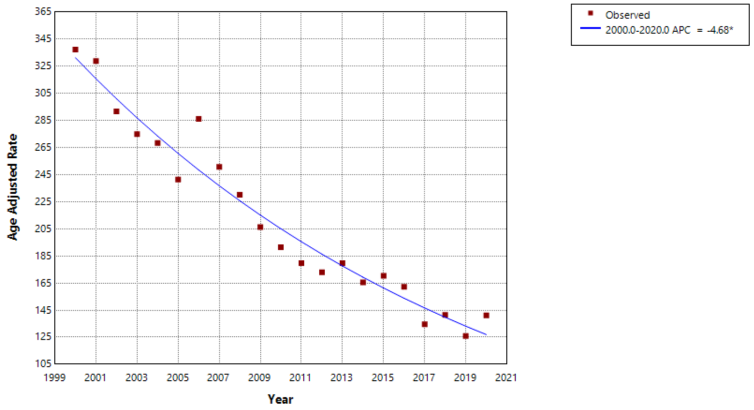

* Indicates that the Annual Percent Change (APC) is significantly different from zero at the alpha = 0.05 level.
Final Selected Model: 0 Joinpoints.

White Population: 0 Joinpoints#

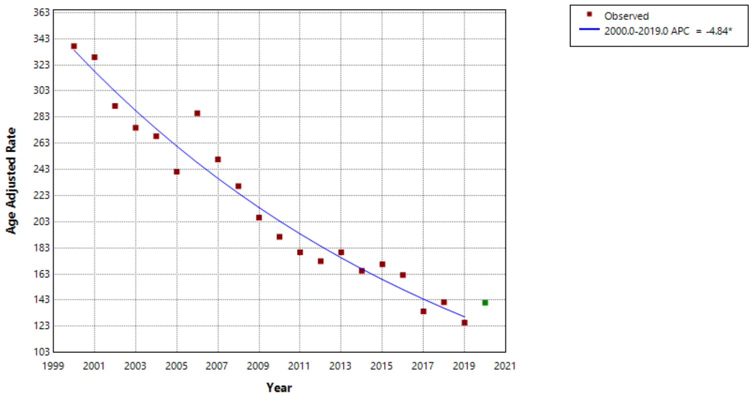

* Indicates that the Annual Percent Change (APC) is significantly different from zero at the alpha = 0.05 level.
# Observed Y Value 2020 was excluded from the model fitting.
Final Selected Model: 0 Joinpoints.

© 2025 Hashemian M et al. *JAMA Network Open*.

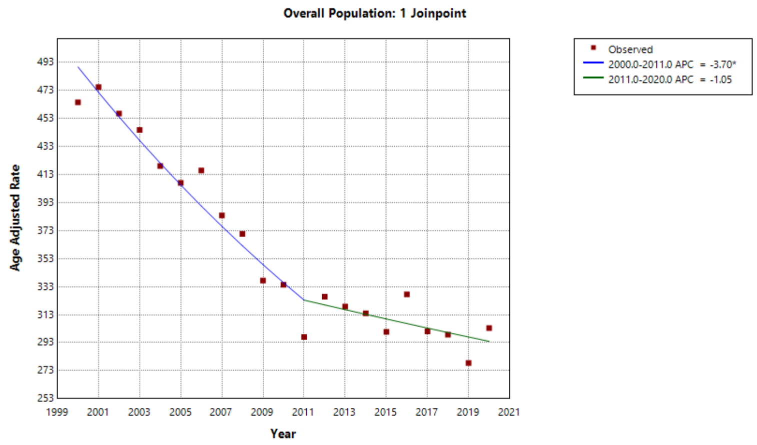

* Indicates that the Annual Percent Change (APC) is significantly different from zero at the alpha = 0.05 level.
Final Selected Model: 1 Joinpoint.

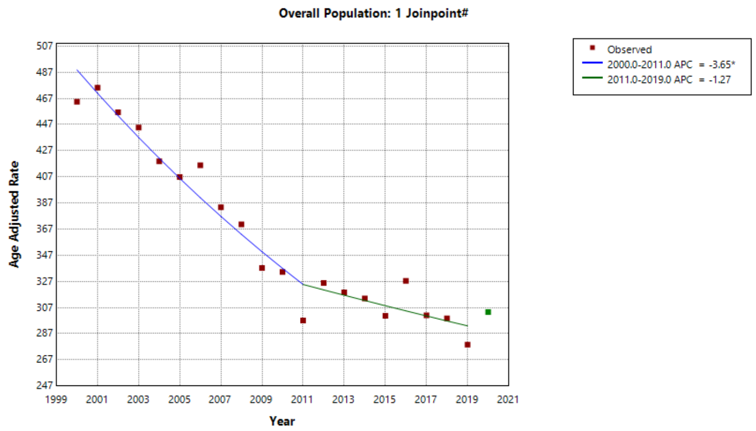

* Indicates that the Annual Percent Change (APC) is significantly different from zero at the alpha = 0.05 level.
# Observed Y Value 2020 was excluded from the model fitting.
Final Selected Model: 1 Joinpoint.

C) *Cancer mortality before (left) and after (right) excluding 2020*

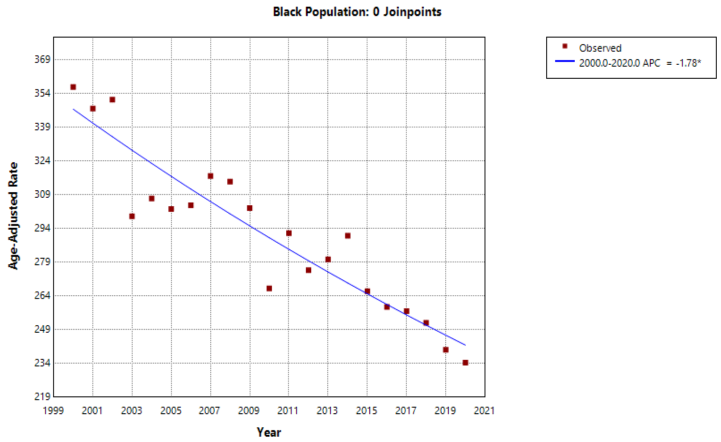

* Indicates that the Annual Percent Change (APC) is significantly different from zero at the alpha = 0.05 level.
Final Selected Model: 0 Joinpoints.

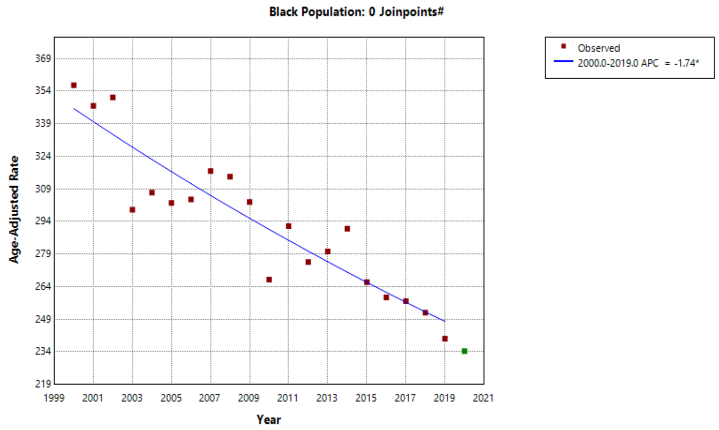

* Indicates that the Annual Percent Change (APC) is significantly different from zero at the alpha = 0.05 level.
# Observed Y Value 2020 was excluded from the model fitting.
Final Selected Model: 0 Joinpoints.

© 2025 Hashemian M et al. *JAMA Network Open*.

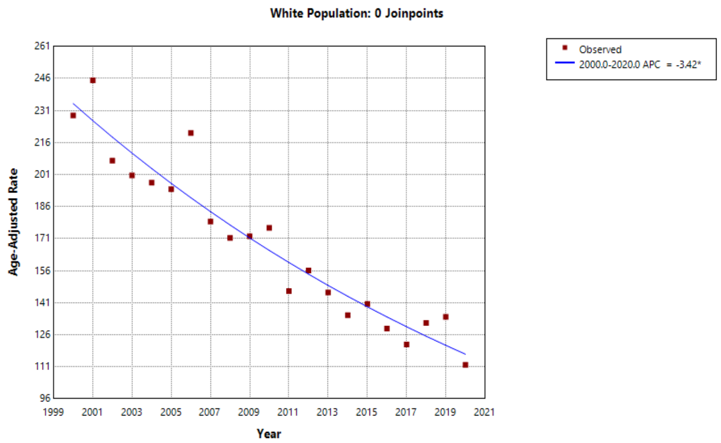

* Indicates that the Annual Percent Change (APC) is significantly different from zero at the alpha = 0.05 level.
Final Selected Model: 0 Joinpoints.

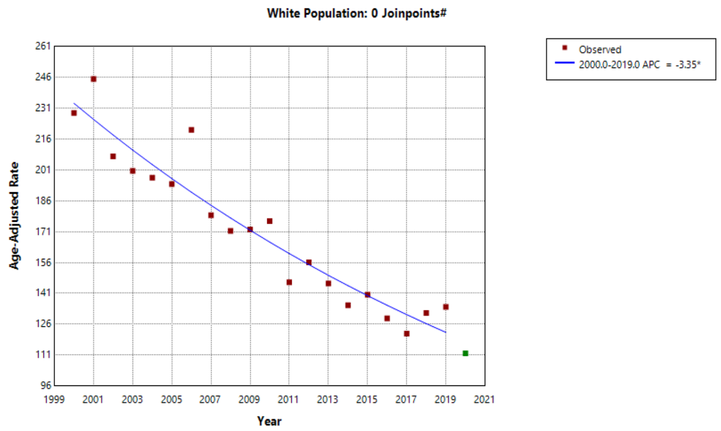

* Indicates that the Annual Percent Change (APC) is significantly different from zero at the alpha = 0.05 level.
# Observed Y Value 2020 was excluded from the model fitting.
Final Selected Model: 0 Joinpoints.

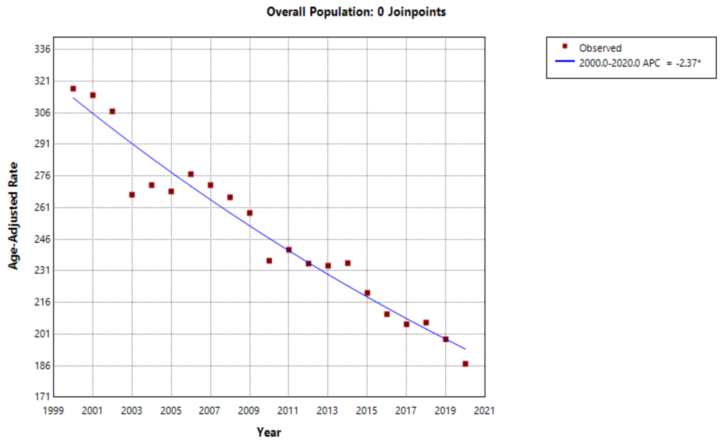

* Indicates that the Annual Percent Change (APC) is significantly different from zero at the alpha = 0.05 level.
Final Selected Model: 0 Joinpoints.

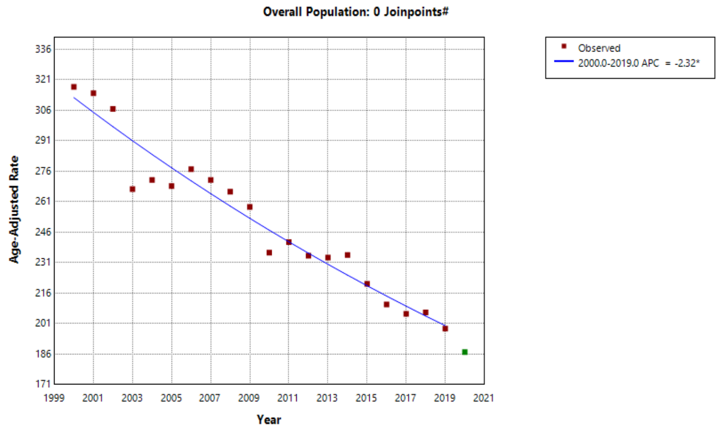

* Indicates that the Annual Percent Change (APC) is significantly different from zero at the alpha = 0.05 level.
# Observed Y Value 2020 was excluded from the model fitting.
Final Selected Model: 0 Joinpoints.

© 2025 Hashemian M et al. *JAMA Network Open*.

eFigure 4, Rate ratios comparing Black individuals to White individuals before and after excluding deaths occurred in 2020

(A) All-cause, CVD, Cancer mortality before (left) and after (right) excluding 2020

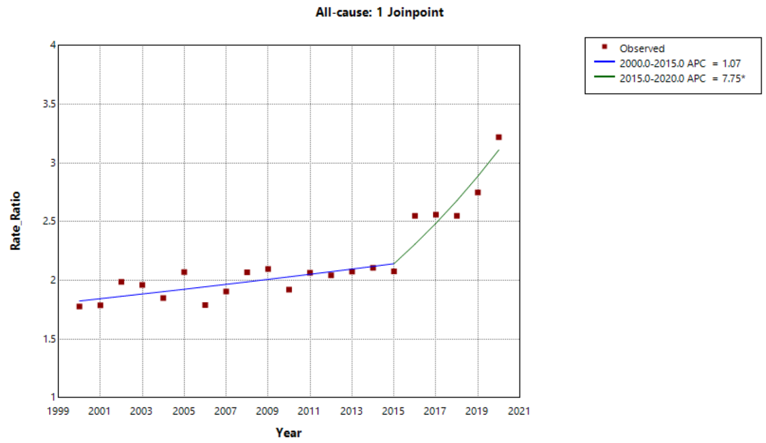

* Indicates that the Annual Percent Change (APC) is significantly different from zero at the alpha = 0.05 level.
Final Selected Model: 1 Joinpoint.

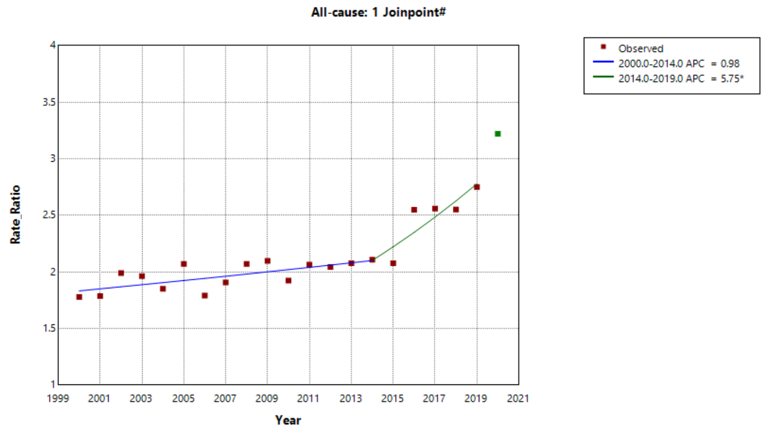

* Indicates that the Annual Percent Change (APC) is significantly different from zero at the alpha = 0.05 level.
\# Observed Y Value 2020 was excluded from the model fitting.
Final Selected Model: 1 Joinpoint.

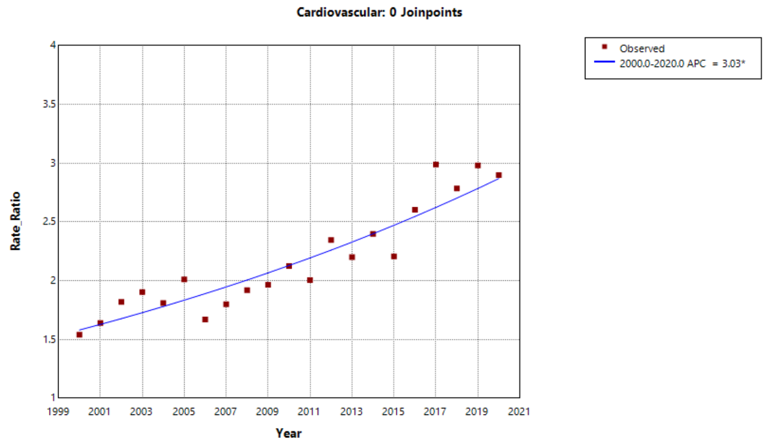

* Indicates that the Annual Percent Change (APC) is significantly different from zero at the alpha = 0.05 level.
Final Selected Model: 0 Joinpoints.

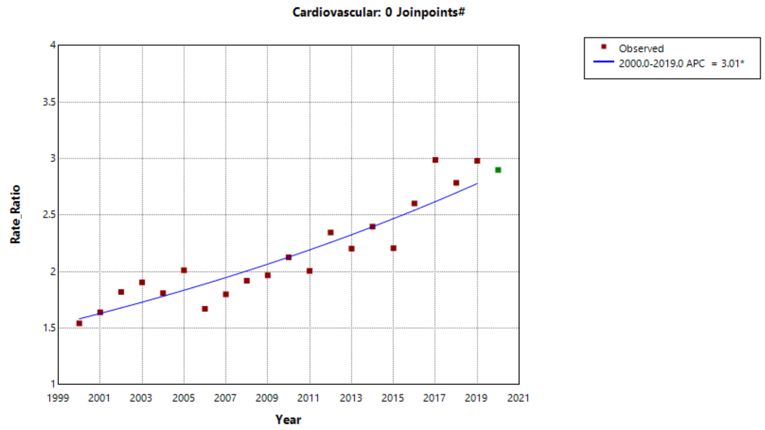

* Indicates that the Annual Percent Change (APC) is significantly different from zero at the alpha = 0.05 level.
\# Observed Y Value 2020 was excluded from the model fitting.
Final Selected Model: 0 Joinpoints.

© 2025 Hashemian M et al. *JAMA Network Open*.

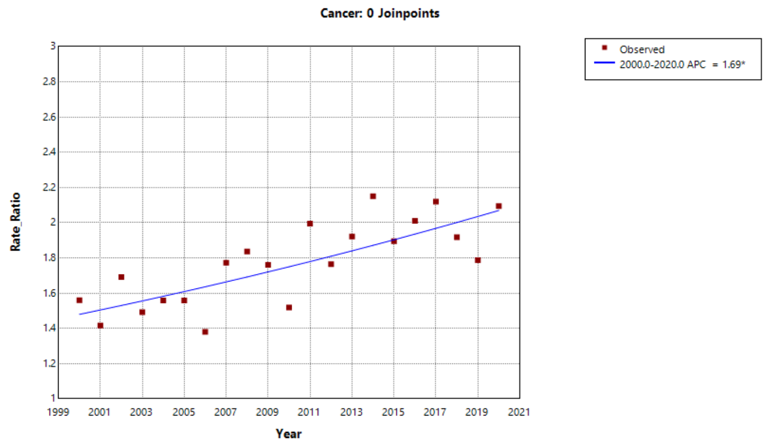

* Indicates that the Annual Percent Change (APC) is significantly different from zero at the alpha = 0.05 level.
Final Selected Model: 0 Joinpoints.

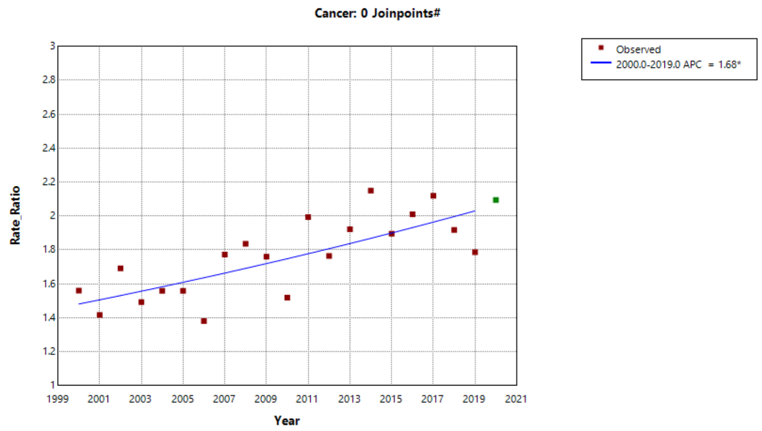

* Indicates that the Annual Percent Change (APC) is significantly different from zero at the alpha = 0.05 level.
# Observed Y Value 2020 was excluded from the model fitting.
Final Selected Model: 0 Joinpoints.

© 2025 Hashemian M et al. *JAMA Network Open*.

eFigure 5, Rate ratios comparing Black individuals to White individuals by sex before and after excluding deaths occurred in 2020

(B) Rate Ratio for All-cause mortality by gender before (left) and after (right) excluding 2020

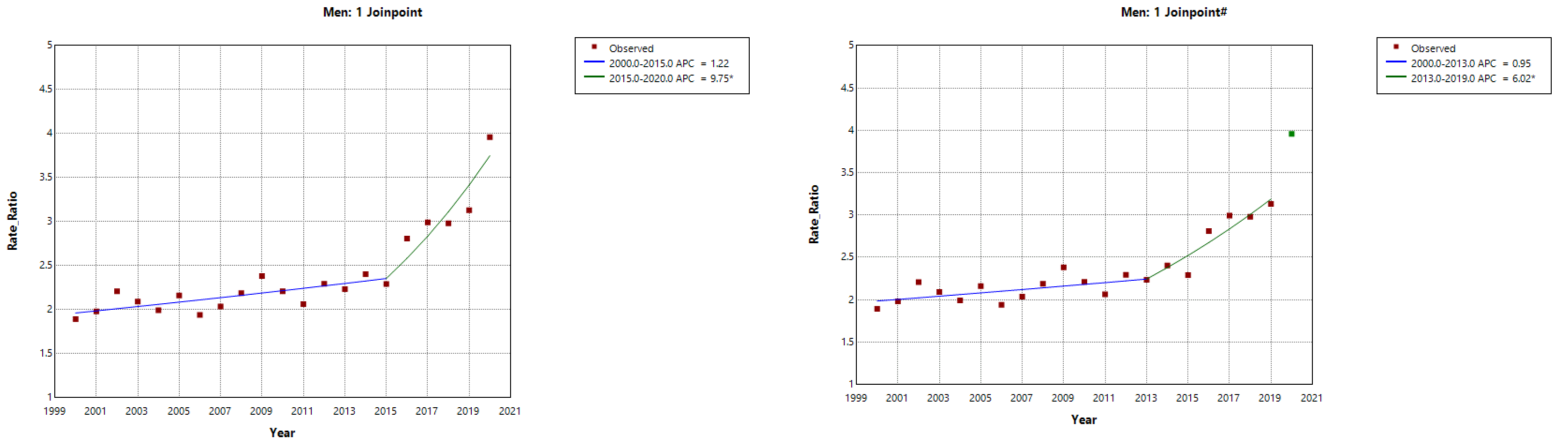

* Indicates that the Annual Percent Change (APC) is significantly different from zero at the alpha = 0.05 level.
Final Selected Model: 1 Joinpoint.

* Indicates that the Annual Percent Change (APC) is significantly different from zero at the alpha = 0.05 level.
\# Observed Y Value 2020 was excluded from the model fitting.
Final Selected Model: 1 Joinpoint.

© 2025 Hashemian M et al. *JAMA Network Open*.

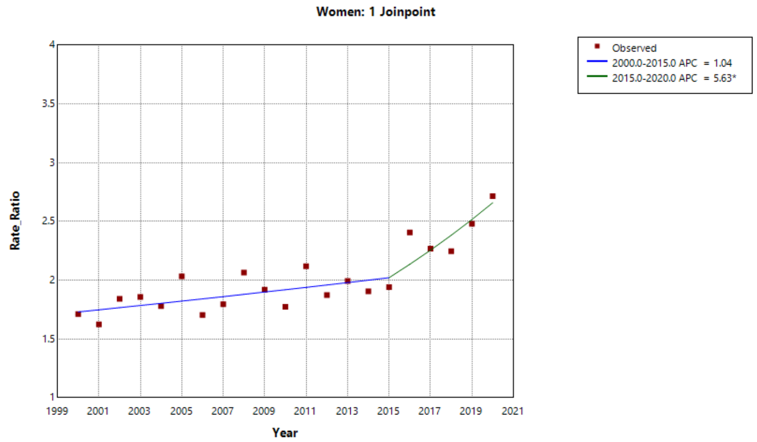

* Indicates that the Annual Percent Change (APC) is significantly different from zero at the alpha = 0.05 level.
Final Selected Model: 1 Joinpoint.

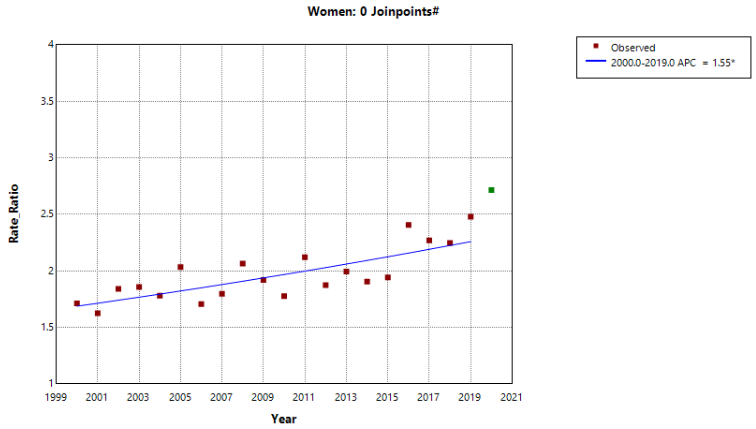

* Indicates that the Annual Percent Change (APC) is significantly different from zero at the alpha = 0.05 level.
\# Observed Y Value 2020 was excluded from the model fitting.
Final Selected Model: 0 Joinpoints.

(C) Rate Ratio for CVD mortality by gender before (left) and after (right) excluding 2020

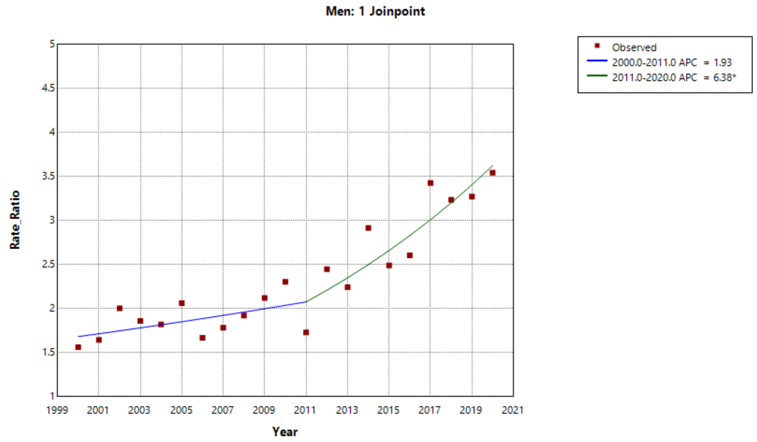

* Indicates that the Annual Percent Change (APC) is significantly different from zero at the alpha = 0.05 level.
Final Selected Model: 1 Joinpoint.

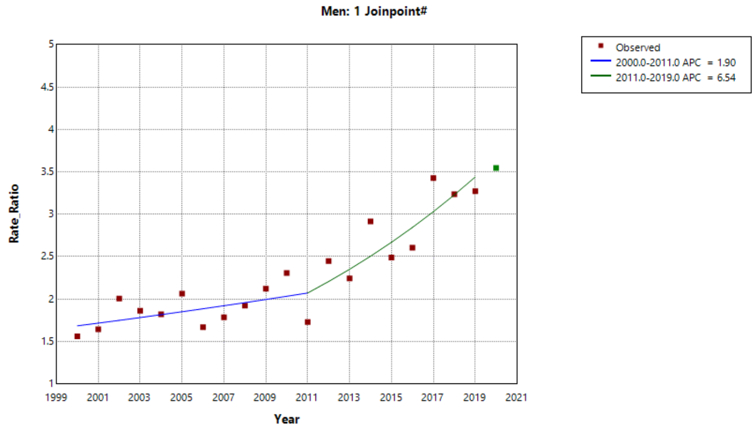

* Indicates that the Annual Percent Change (APC) is significantly different from zero at the alpha = 0.05 level.
\# Observed Y Value 2020 was excluded from the model fitting.
Final Selected Model: 1 Joinpoint.

© 2025 Hashemian M et al. *JAMA Network Open*.

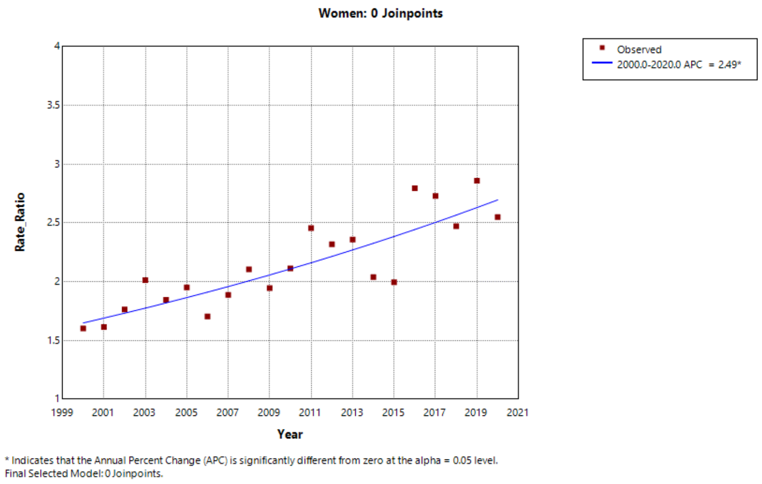

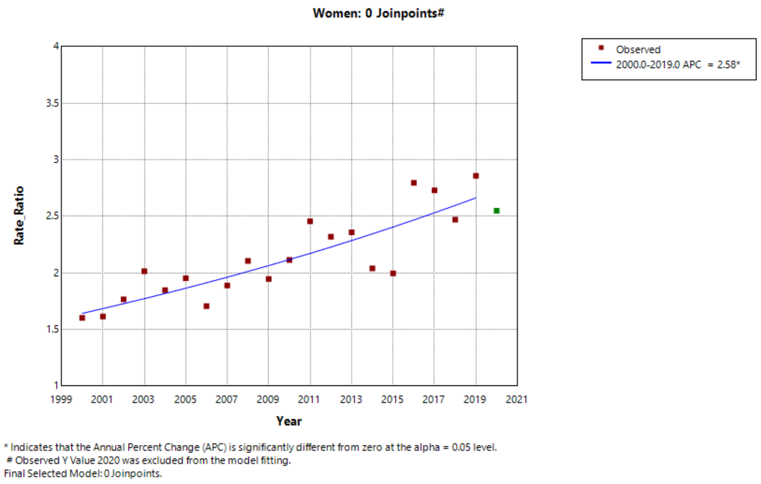

(C) Rate Ratio for Cancer mortality by gender before (left) and after (right) excluding 2020

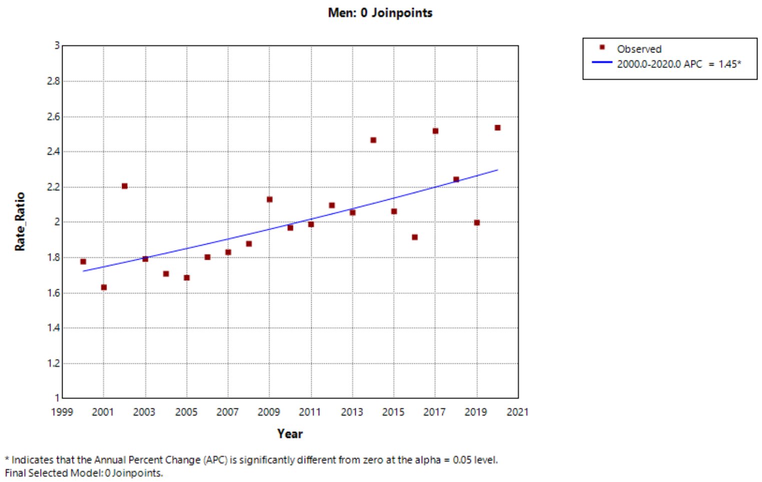

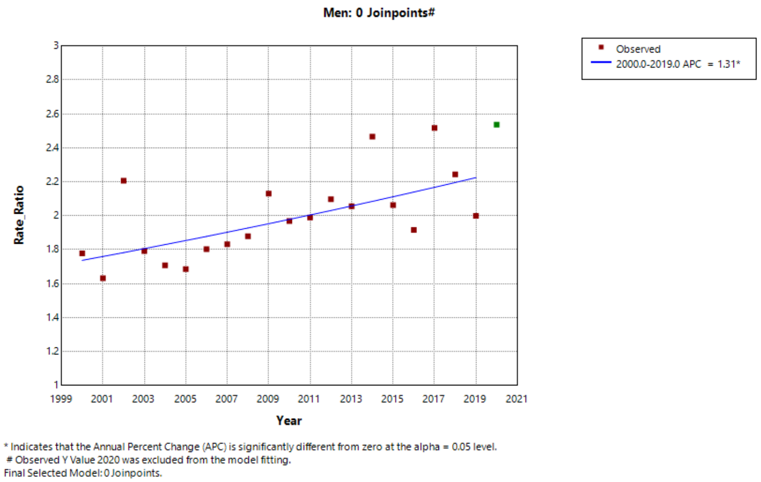

© 2025 Hashemian M et al. *JAMA Network Open*.

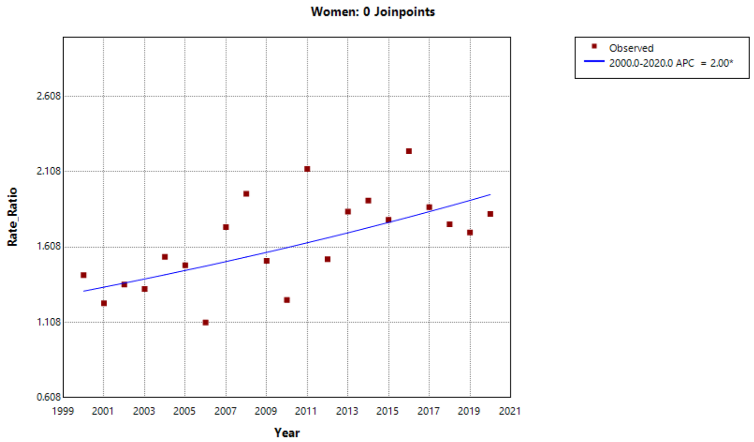

* Indicates that the Annual Percent Change (APC) is significantly different from zero at the alpha = 0.05 level.
Final Selected Model: 0 Joinpoints.

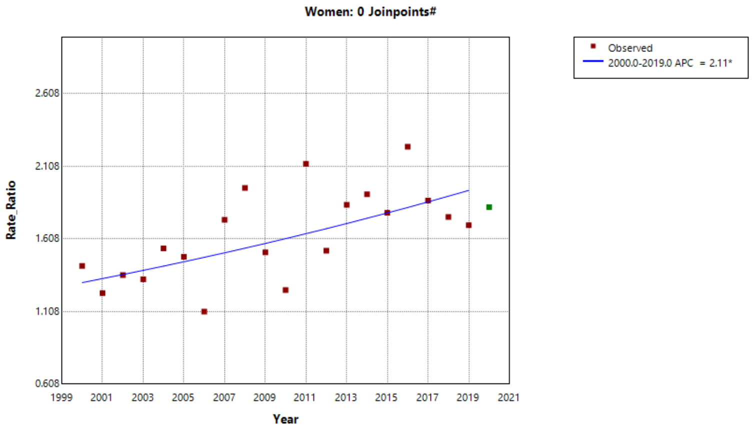

* Indicates that the Annual Percent Change (APC) is significantly different from zero at the alpha = 0.05 level.
# Observed Y Value 2020 was excluded from the model fitting.
Final Selected Model: 0 Joinpoints.

© 2025 Hashemian M et al. *JAMA Network Open*.

eFigure 6, Joinpoint analysis without (top) and with (bottom) considering autocorrelated errors for age-adjusted mortality rate in Black, White, and overall population after excluding deaths occurred in 2020.

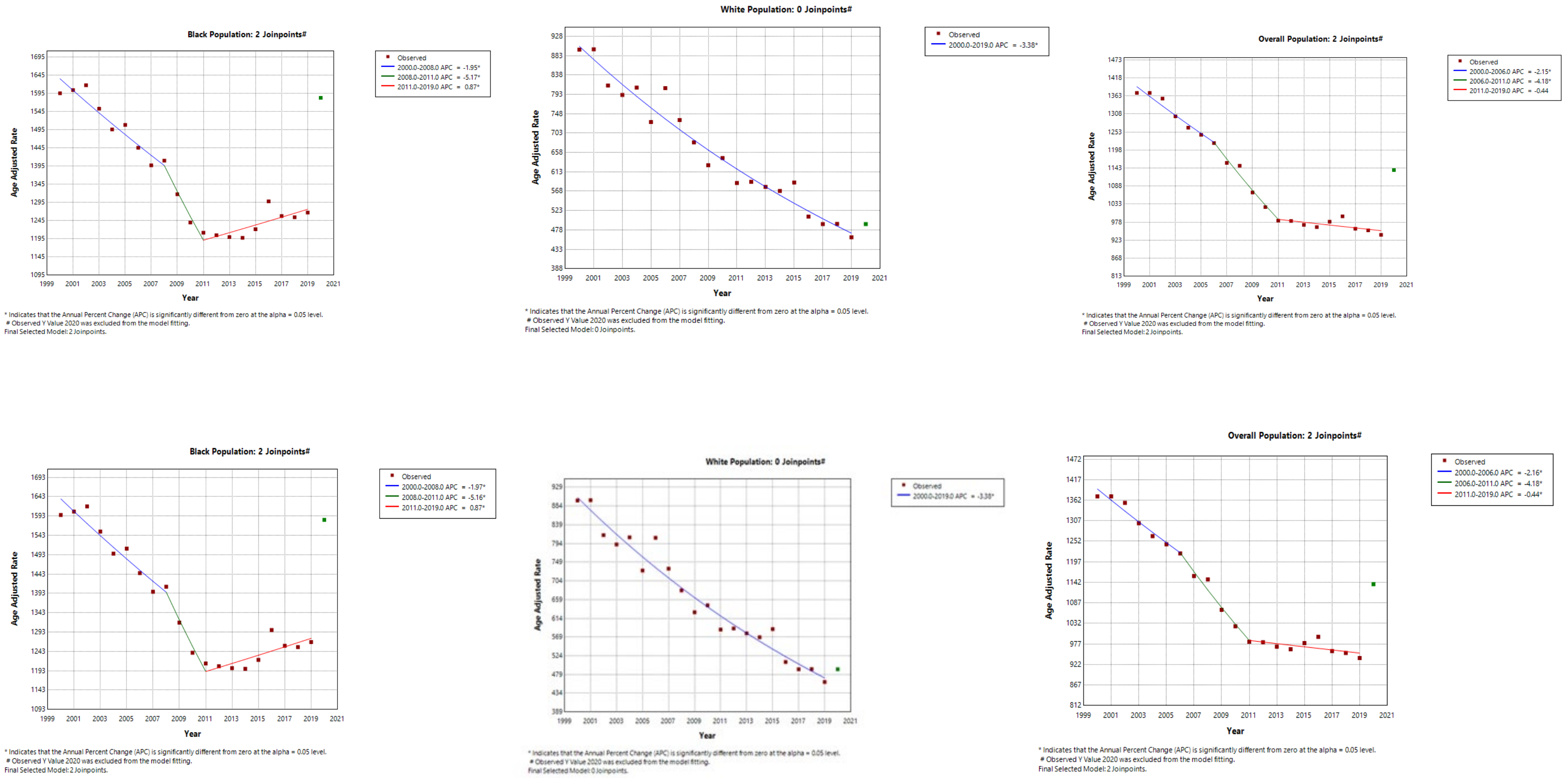

© 2025 Hashemian M et al. *JAMA Network Open*.
