# Supplementary material for: Demographic Differences in Mortality in the District of Columbia
Source: JAMA Netw Open. 2025 Mar 28;8(3):e252290. doi: 10.1001/jamanetworkopen.2025.2290 (PMC11953761; doi:10.1001/jamanetworkopen.2025.2290)
Supplement: Supplement 2. — Data Sharing Statement [file jamanetwopen-e252290-s002.pdf]

Data Sharing Statement

Hashemian. Racial Disparities in Mortality in the District of Columbia. *JAMA Netw Open*. Published online March 28, 2025. doi:

Data
Data available: No

Additional Information
Explanation for why data not available: public available data
